# A Novel Polysilicon-Fill-Strengthened Etch-Through 3D Trench Electrode Detector: Fabrication Methods and Electrical Property Simulations

**DOI:** 10.3390/mi16080912

**Published:** 2025-08-06

**Authors:** Xuran Zhu, Zheng Li, Zhiyu Liu, Tao Long, Jun Zhao, Xinqing Li, Manwen Liu, Meishan Wang

**Affiliations:** 1 School of Integrated Circuits, Ludong University, Yantai 264025, China; 2 Engineering Research Center of Photo Detector Special Chip in Universities of Shandong, Ludong University, Yantai 264025, China; 3 School of Materials Science and Engineering, Xiangtan University, Xiangtan 411105, China; 4 Institute of Microelectronics, Chinese Academy of Sciences, Beijing 100029, China

**Keywords:** novel polysilicon-fill-strengthened etch-through 3D trench electrode, full 3D simulation, electric field distribution, electric potential distribution, fully depleted voltage, induced transient current

## Abstract

Three-dimensional trench electrode silicon detectors play an important role in particle physics research, nuclear radiation detection, and other fields. A novel polysilicon-fill-strengthened etch-through 3D trench electrode detector is proposed to address the shortcomings of traditional 3D trench electrode silicon detectors; for example, the distribution of non-uniform electric fields, asymmetric electric potential, and dead zone. The physical properties of the detector have been extensively and systematically studied. This study simulated the electric field, potential, electron concentration distribution, complete depletion voltage, leakage current, capacitance, transient current induced by incident particles, and weighting field distribution of the detector. It also systematically studied and analyzed the electrical characteristics of the detector. Compared to traditional 3D trench electrode silicon detectors, this new detector adopts a manufacturing process of double-side etching technology and double-side filling technology, which results in a more sensitive detector volume and higher electric field uniformity. In addition, the size of the detector unit is 120 µm × 120 µm × 340 µm; the structure has a small fully depleted voltage, reaching a fully depleted state at around 1.4 V, with a saturation leakage current of approximately $4.8\times 10^{-10}A$, and a geometric capacitance of about 99 fF.

## 1. Introduction

At present, as society continues to progress and science and technology keep advancing, the application of semiconductor materials has become an indispensable part of life. The detector technology based on semiconductor materials has emerged and matured. Among various types of semiconductor detectors, silicon detectors have many advantages, such as high energy and position resolution, fast response speed, high sensitivity, and ease of integration. They have been widely used in fields such as high-energy physics [1,2,3], astrophysics, nuclear medicine [4,5], X-ray imaging [6], national defense, industry [7], high-energy physics experiments in high-energy particle detection, and many others. The commonly used thickness of silicon detectors is about 300 µm. With the development of particle physics and related fields, research on silicon detectors will continue to be carried out both domestically and internationally.

The common types of silicon detectors mainly include silicon microstrip detectors [8,9], silicon drift detectors [10,11], three-dimensional columnar electrode silicon detectors [12,13,14], and three-dimensional trench electrode silicon detectors [15,16], which work in a similar way. With the evolution and development of various types of semiconductor detectors from 2D to 3D, the structure and performance of detectors are constantly being improved and optimized, and 3D silicon detectors have been proven to be more radiation resistant than silicon planar detectors [13]. Although a double-sided detector concept was proposed in Ref. [16] as well, no details were provided on the detector structures and processing technologies. The first non-etch-through 3D trench electrode detector was first fabricated and tested in CNM, with the results reported in Ref. [16].

The trench wall electrode (straight) concept, initially proposed by C. Kenney [17], employs embedded electrode structures within silicon substrates. This technologically demanding approach requires advanced design methodologies and precision manufacturing techniques. In 1997, S.Parker designed and developed a structure completely different from planar detectors, called a three-dimensional columnar electrode silicon detector. In 2005, Brookhaven Laboratory in the United States proposed a new type of 3D electrode detector, the 3D trench electrode silicon detector. The electric field distribution within the detector unit of this trench-shaped electrode is more uniform, and the isolation of each unit is stronger when forming an array. But for traditional 3D trench electrode silicon detectors, it is necessary to leave an un-etched thickness to ensure that the detector body does not detach from the substrate during the etching process. After etching, due to the thin un-etched thickness of the silicon substrate, the trench electrode cannot completely penetrate the entire silicon substrate and usually leaves about 10 percent of the depth of the silicon substrate. The un-etched substrate here is the dead zone, which is a weak or zero-electric-field region that exists in the detector [18]. If the dead zone occupies a large part of the detector volume, the electrical characteristics of the detector will be severely affected, thereby reducing the charge collection efficiency.

In order to address the aforementioned shortcomings of traditional 3D trench electrode silicon detectors, this paper proposes a novel polysilicon-fill-strengthened etch-through 3D trench electrode detector and systematically studies its electrical properties. This new type of detector is etched and filled on both sides of the silicon detector to form a through trench electrode, which is embedded in the silicon body. Its depletion electric field is perpendicular to the thickness direction of the silicon wafer, so its depletion voltage is independent of thickness. Each pixel unit in the detector is isolated from other pixel units by highly doped trenches, making it an electrically independent unit. And adjacent detector units can also share the same trench electrode, which greatly reduces dead zones and electrode connections. In this article, the simulation assistance software was used for electrical simulation and performance comparison of the detector.

## 2. The Structure of a Novel Polysilicon-Fill-Strengthened Etch-Through 3D Trench Electrode Detector on N-Type Bulk

### 2.1. The Model of a Novel Polysilicon-Fill-Strengthened Etch-Through 3D Trench Electrode Detector

**Figure 1:**
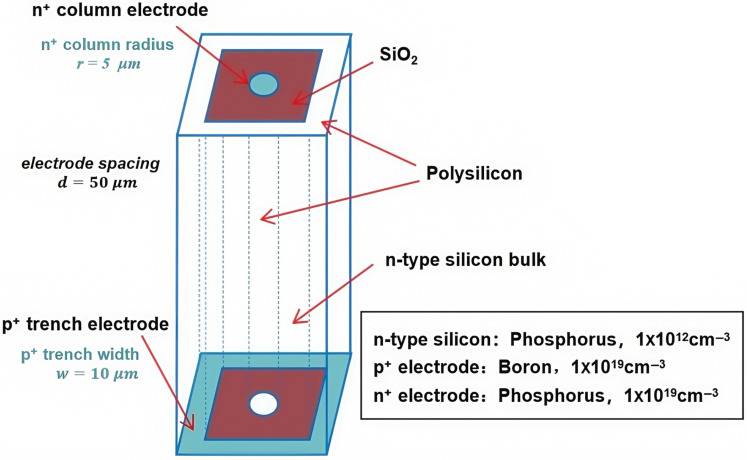
A novel polysilicon-fill-strengthened etch-through 3D trench electrode detector structure.

The most obvious advantage of 3D electrode detectors is its radiation hardness, which has wide applications in high-energy physics experiments. In this work, however, we are focusing our detector in the area of X-ray detection and imaging with little radiation effect. Therefore, we can use a relatively large detector size and thickness in the realm of hundreds of microns. When selecting a square as the shape of the detector unit, the practical advantage is that it can easily generate a detector array, and the resulting pixel detector also has a regular shape that can be easily cut from the chip and placed in the application device. The detector bulk is N-type Si with $1\times 10^{12}/{\mathrm{cm}}^{3}$ phosphorus doping with a thickness of 340 µm. The central collection column electrode is doped with $n^{+}$ at a concentration of $1\times 10^{19}/{\mathrm{cm}}^{3}$, and the 3D trench electrode is doped with $p^{+}$ at a concentration of $1\times 10^{19}/{\mathrm{cm}}^{3}$. The p-n junction is placed near the 3D trench electrode to ensure good electric field distribution and low full depletion voltage of the detector. At the top of the detector, the center column electrode is covered with a 1 µm layer of aluminum, and the area between the center column electrode and the etched trench electrode is covered with a 1 µm layer of silicon oxide. At the bottom of the detector, the trench electrode is covered with a 1 µm layer of aluminum, and the gap between the trench electrode and the etched columnar region is covered with a 1 µm layer of silicon oxide. The trench and column are filled with polysilicon. Figure 1 shows a schematic diagram of the structure of a novel polysilicon-fill-strengthened etch-through 3D trench electrode detector.

### 2.2. The Technological Process of a Novel Polysilicon-Fill-Strengthened Etch-Through 3D Trench Electrode Detector

The primary technical challenge in fabricating 3D electrode detectors is how to form precisely trench-shaped conductive elements. In 2025, Liu, M. and others proposed and simulated a double-sided 3D trench electrode detector structure; in the simulation, the wafer thickness of the detector unit was set to 700 µm, and the width of the column electrode and trench electrode were the same, both 5 µm. The dark current of this structure was about $10^{-10}A$, and the capacitance was less than 250 fF. The acquisition time was on the order of tens of nanoseconds. Through Bosch deep reactive ion etching (DRIE), the thickness of the 3D trench electrode can reach 311 µm, and the aspect ratio can reach 105:1 [19], with a precision of double-sided alignment better than 2 µm. Since our trench width is 10 µm, this allows an acceptable alignment of the top and bottom trenches, thereby enabling practical implementation of such advanced devices. Since, during the process of trench filling of polysilicon, the top and bottom surface will be protected, there will be no polysilicon layers formed on both surfaces, and therefore there will be no bowing/warping effects.

**Figure 2:**
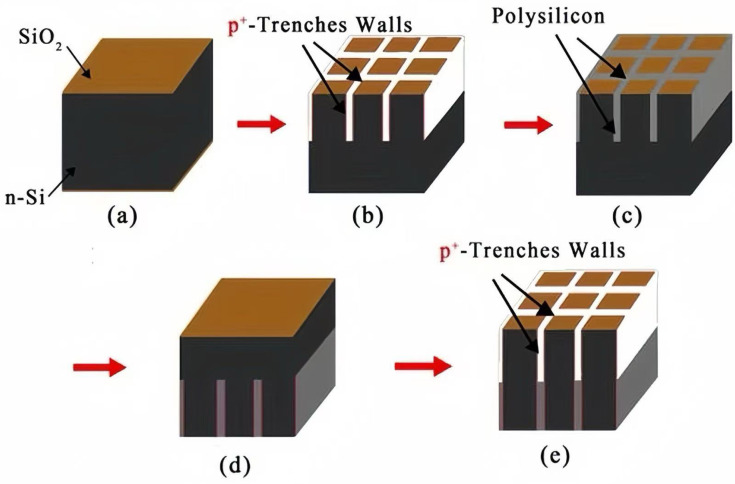
Novel polysilicon-fill-strengthened etch-through 3D trench electrode detector fabrication process steps (part one).

**Figure 3:**
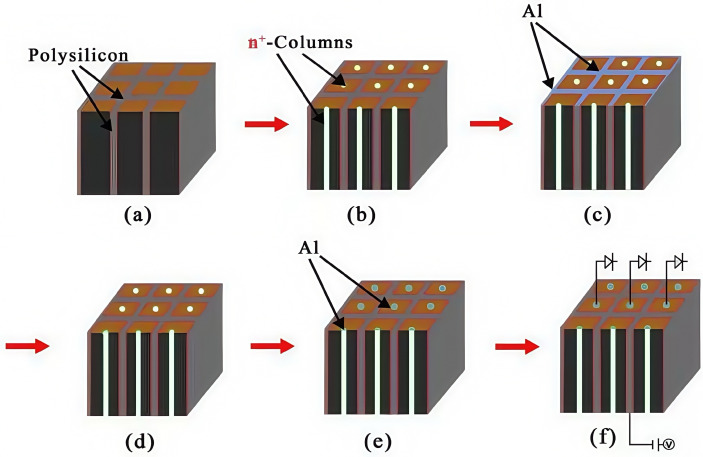
Novel polysilicon-fill-strengthened etch-through 3D trench electrode detector fabrication process steps (part two).

Taking a 3 × 3 detector array as an example, we will introduce the manufacturing method of the detector here. The majority charge carrier in N-type silicon is electron, with an electron mobility of approximately 1450 cm^2^/s/V, significantly higher than the mobility of holes in P-type silicon, which is 450 cm^2^/s/V. However, for applications in high-radiation environments, it is recommended to use a P-type substrate configuration for its radiation hardness [20]. In this case, the peripheral trenches of the device need to be heavily doped with $N^{+}$ (Phosphorus), and the central columnar electrode doped with $P^{+}$ (Boron), which becomes a complicated P-type process. Therefore, for our cases of non-irradiation or light irradiation conditions, a lightly doped N-type silicon substrate is preferred for simplicity in detector process. As shown in Figure 2, the first part of the detector fabrication process steps is as follows: (a) select lightly doped N-type silicon, and cover the upper and lower surfaces with 1 µm of SiO_2_ (thermally grown SiO_2_); (b) etch half of the silicon body to form a trench electrode of a depth of half of the wafer and a width of 5–20 µm, doping the trench walls with $P^{+}$ (Boron) by diffusion or ion implantation (with a special angle related to the trench depth and width), with P-type heavy doping at a doping concentration of $1\times 10^{18}/{\mathrm{cm}}^{3}$–$2\times 10^{20}/{\mathrm{cm}}^{3}$; (c) conduct polysilicon filling, where the polysilicon deposition method can be chemical vapor deposition (CVD); (d) flip over the wafer to the bottom side; and (e) for $P^{+}$-trench wall etching, dope the trench walls with $P^{+}$ (Boron) by diffusion or ion implantation, with P-type heavy doping at a doping concentration of $1\times 10^{18}/{\mathrm{cm}}^{3}$–$2\times 10^{20}/{\mathrm{cm}}^{3}$. Figure 3 shows the second part of the detector fabrication process steps as follows: (a) polysilicon filling; (b) $N^{+}$-column etching, doping the columns with $N^{+}$ (Phosphorus), with N-type heavy doping at a doping concentration of $1\times 10^{18}/{\mathrm{cm}}^{3}$–$2\times 10^{20}/{\mathrm{cm}}^{3}$; (c) Al-coating on $P^{+}$-trench with a thickness of 1 µm (trench electrodes will be isolated by the negative surface charge on the silicon surface induced by positive oxide charge); (d) flip over the wafer to the top side; (e) Al-coating on the top side ($N^{+}$-columns are isolated by the surrounding $P^{+}$ trenches); and (f) connection to readout electronics. A SiO_2_ insulating layer isolates the anode and cathode, exhibiting an initial oxide charge density of $4\times 10^{11}/{\mathrm{cm}}^{2}$. This value demonstrates a positive correlation with radiation intensity, potentially escalating under prolonged exposure.

## 3. Electrical Characteristic Results

### 3.1. Three-Dimensional Simulation of a Novel Polysilicon-Fill-Strengthened Etch-Through 3D Trench Electrode Detector

**Figure 4:**
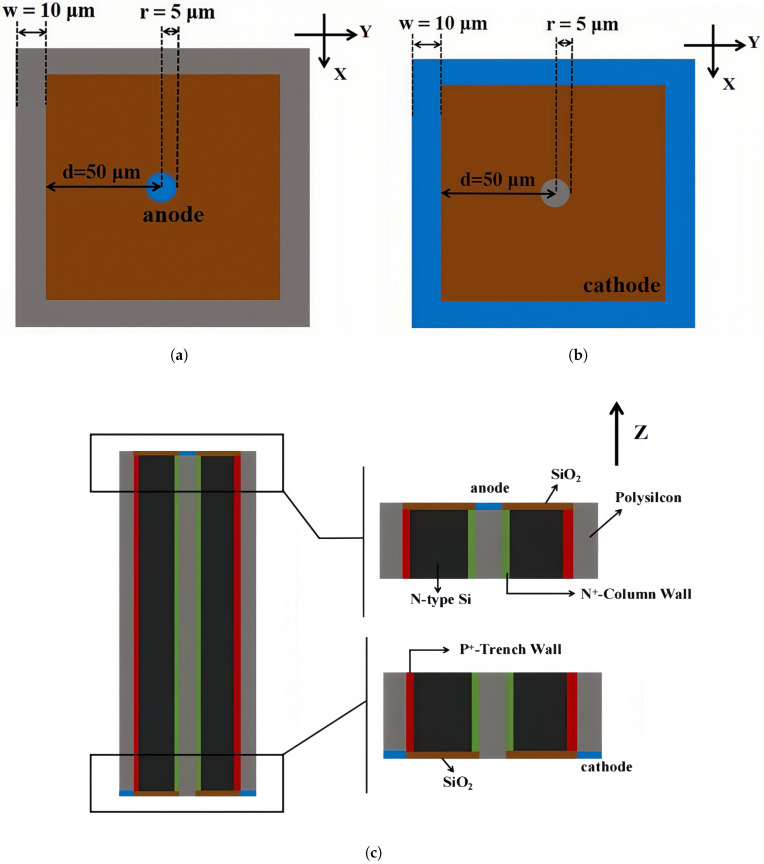
Structure of novel polysilicon-fill-strengthened etch-through 3D trench electrode detector structure. The axis units are micrometers. The directions of observation here are the (**a**) top side, (**b**) bottom side, and (**c**) sectional view.

This part of the simulation was conducted using the full 3D simulation assistance software, which can accurately simulate complex three-dimensional structures and their electrical properties. The physical models used in our simulations include SRH recombination, doping-dependent carrier mobility (Masetti model), and high field saturation effect. TCAD contains many comprehensive components that users can directly call as needed. Components have Sentaurus Workbench, Sentaurus Device, Sentaurus Process, Sentaurus Structure Editor, Mesh and Noffset 3D, Ligament, Tecplot SV, Inspect, and Calibration Kit Wait. The fixed charge density of the oxide layer was set to be $4\times 10^{11}/{\mathrm{cm}}^{2}$ to simulate the influence of oxide charge. Figure 4a and Figure 4b, respectively, present schematic diagrams of the upper and lower surfaces of the detector unit, visually demonstrating the positions and dimensions of the anode and cathode. In our study, we chose a detector unit cell with the following parameters: cell size 110 µm, trench width 10 µm, central column diameter 10 µm, and detector thickness 340 µm. In this case, the relative inactive volume due to trenches and column is 18%. We note that if we increased cell size, this relative inactive volume was greatly reduced. For example, if we increased cell size to 170 µm, the relative inactive volume will be reduced to 11.7%. Figure 4c provides a cross-sectional view of the structure, with axial units in micrometers (µm). By means of the cross-sectional view, the penetration of the trench electrode and the distribution of material layers can be more intuitively observed, providing important references for subsequent performance analysis and optimization. A negative bias voltage is applied to the cathode electron on the bottom side, and the anodes on the top side are connected to the external readout electronics that are at virtual ground (0 V).

### 3.2. Full Depletion Voltage and Electric Potential Distribution of a Novel Polysilicon-Fill-Strengthened Etch-Through 3D Trench Electrode Detector

In the novel polysilicon-fill-strengthened etch-through 3D trench electrode detector, the N-type silicon bulk has a doping concentration of $1\times 10^{12}/{\mathrm{cm}}^{3}$, so the effective doping concentration $N_{eff}$ = $1\times 10^{12}/{\mathrm{cm}}^{3}$. The detector thickness is chosen as 340 µm.

According to the full depletion voltage formula of traditional 3D trench electrode detectors [12]:

(1) $$V_{fd}=\frac{{eN}_{eff}}{4\epsilon_{Si}\epsilon_{0}}{\;d}^{2}+V_{bi}$$

where *e* is the electronic charge, *e* = $1.6\times 10^{19}\;C$; $\epsilon_{0}$ is the vacuum dielectric constant, $\epsilon_{0}$ = $8.854\times 10^{-12}\;F/m$; $\epsilon_{Si}$ is the relative dielectric constant of silicon, $\epsilon_{Si}$ = 11.9; *d* is the electrode spacing, *d* = 45 µm; and $V_{bi}$ is the built-in potential of the p-n junction in silicon materials, $V_{bi}$≈ 0.75 V. We could obtain a full depletion voltage $V_{fd}$≈ 1.5 V, so the depletion voltage of our novel polysilicon-fill-strengthened etch-through 3D trench electrode detector should be around 1.5 V, which is close to our previous value of 1.4 V obtained from simulations.

**Figure 5:**
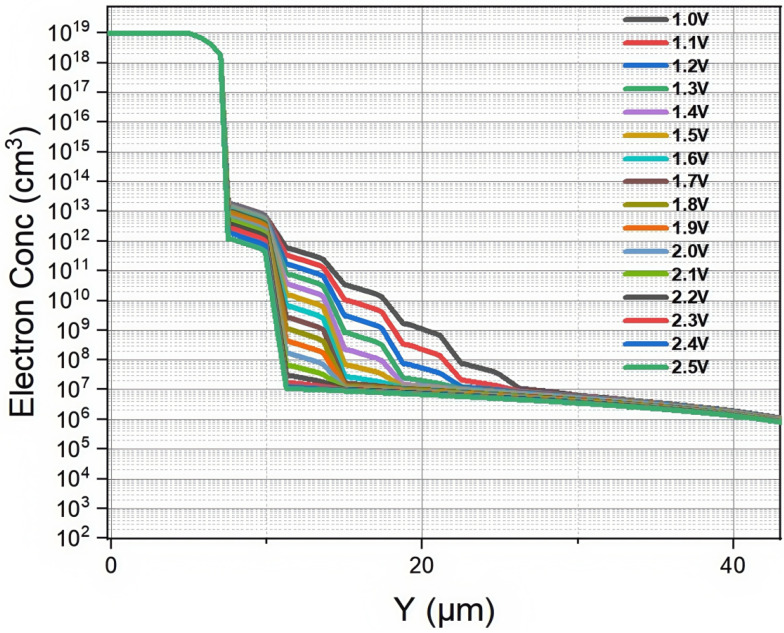
Partial plot of the 2D electron concentration curves of a novel polysilicon-fill-strengthened etch-through 3D trench electrode detector under different voltages.

The electron concentration distribution obtained from the simulation is presented in Figure 5. The direction of the Y-axis in Figure 5 is perpendicular to the direction of the cylindrical anode. When the bias voltage increases, the electron concentration in the detector gradually decreases until it is completely depleted. Due to the N-type heavy doping of the silicon body around the columnar electrode at the center of the detector unit, the electron concentration at this location shows a stepped upward trend along the Y-axis under different bias voltages. The simulation results show that when the bias voltage rises to 1.4 V, the carrier concentration in the active region of the detector decreases to $1\times 10^{12}/{\mathrm{cm}}^{3}$ below the intrinsic concentration of the silicon substrate. Therefore, we can conclude that the full depletion voltage of the detector is about 1.4 V, which has a small error compared to the result we calculated using the formula for full depletion voltage, which is about 1.5 V.

**Figure 6:**
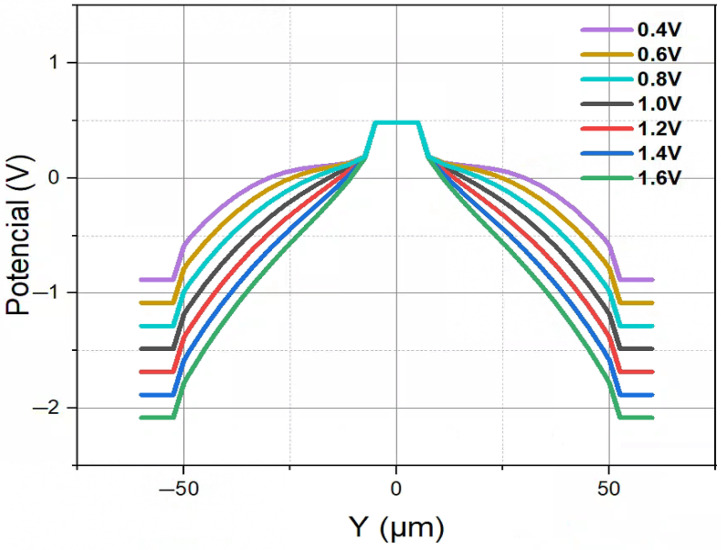
One-dimensional electric potential distribution of a novel polysilicon-fill-strengthened etch-through 3D trench electrode detector under different voltages.

Figure 6 illustrates the electric potential distribution for the novel polysilicon-fill-strengthened etch-through 3D trench electrode detector at different bias voltages. As shown by the Figure 6, the detector exhibits a highly symmetrical electric potential distribution. To establish reverse biasing conditions and facilitate the drift and collection of free electrons at the anode, the system employs maximum (at or over full depletion voltage) positive electrical potential at the central column electrode of the detector.

### 3.3. Electric Field Distribution of a Novel Polysilicon-Fill-Strengthened Etch-Through 3D Trench Electrode Detector

**Figure 7:**
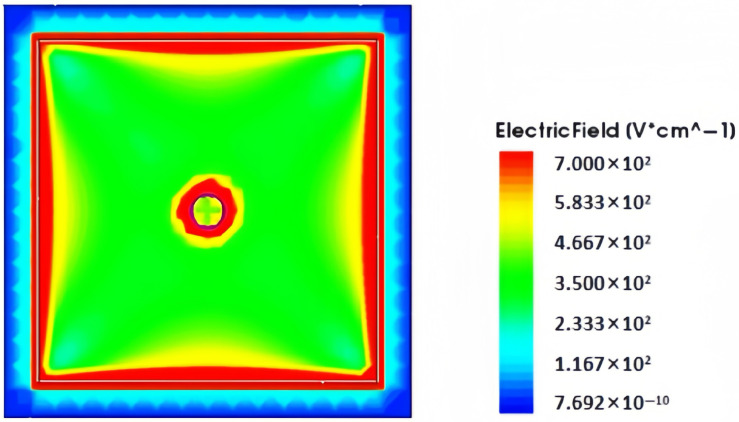
At a bias voltage of 1.4 V, when Z = 170 µm, the electric field distribution of a novel polysilicon-fill-strengthened etch-through 3D trench electrode detector along the Z-axis is presented.

The electric field distribution is an important characteristic of the novel polysilicon-fill-strengthened etch-through 3D trench electrode detector. Figure 7 shows the distribution of the electric field inside the detector along the Z-axis direction at a bias voltage of 1.4 V and Z = 170 µm. It can be seen from the figure that the electric field strength is highest in the central area of the detector, lower in the edge area, and gradually decreases from the center to the outside. The electric field distribution is symmetrical, and the electric field strength in the central area is relatively uniform.

**Figure 8:**
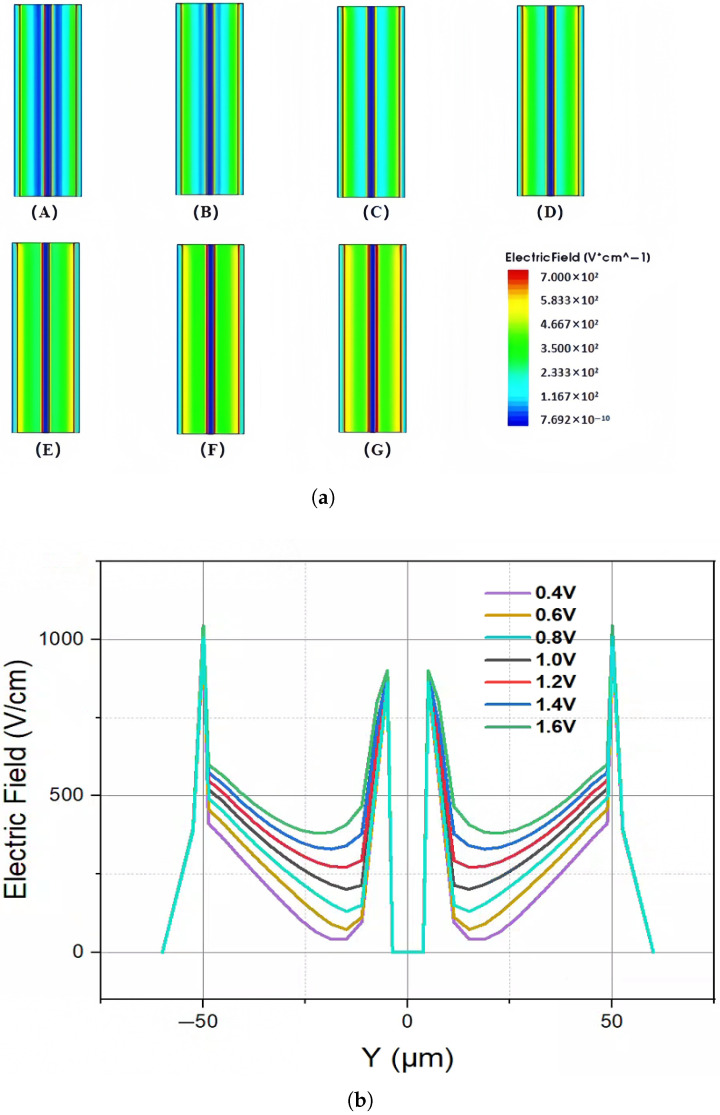
The (**a**) 2D and (**b**) 1D electric field distribution of a novel polysilicon-fill-strengthened etch-through 3D trench electrode detector under different voltages: (**A**) 0.4 V, (**B**) 0.6 V, (**C**) 0.8 V, (**D**) 1.0 V, (**E**) 1.2 V, (**F**) 1.4 V, (**G**) 1.6 V.

We established a 2D cross-section at X = 0 to more clearly display the electric field distribution inside the detector. The simulation results depicting the 2D and 1D electric field distributions of the novel polysilicon-fill-strengthened etch-through 3D trench electrode detector under different bias voltages are illustrated in Figure 8a,b. According to the analysis of the graphical results, the low electric field region within the detector gradually diminishes as the bias voltage increases. When the applied voltage reaches the full depletion voltage, the internal electric field fully covers the entire detector, and the original zero-field region completely disappears. Based on the data analysis from Figure 8, it can be determined that the full depletion voltage of this detector is approximately 1.4 V, which is consistent with the analysis of the changes in electron concentration inside the detector mentioned above. In addition, it can also be clearly seen that the electric field distribution in the depleted area of the detector is extremely uniform, ensuring good charge collection throughout the entire effective detector body.

**Figure 9:**
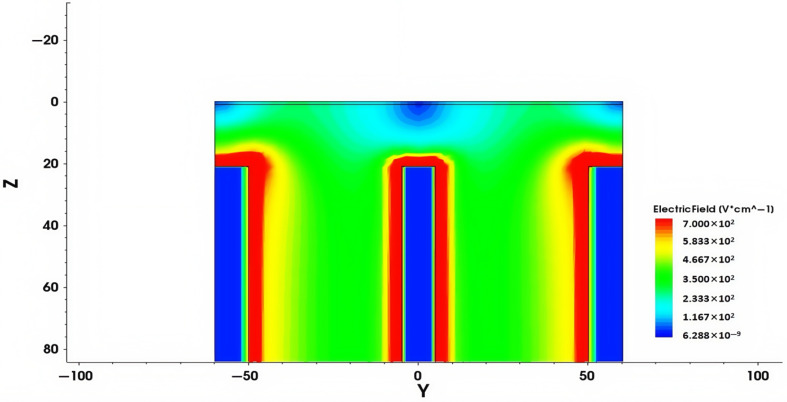
Two-dimensional electric field distribution of traditional 3D trench electrode silicon detector under a bias voltage of 1.4 V.

Figure 9 shows the electric field distribution of a traditional 3D trench electrode silicon detector with the same size. The electrode spacing of the traditional 3D trench detector is 50 µm and the un-etched substrate thickness is 20 µm. Comparative analysis shows that there is significant non-uniformity of the electric field in the un-etched substrate volume at the bottom of traditional 3D trench electrode silicon detectors. In contrast, the new detector achieves excellent electric field uniformity throughout the entire effective volume, which is achieved through symmetrical trench etching and optimized electrode filling. This structure ensures a uniform carrier transport path, thereby improving the charge collection efficiency of the detector in high-precision detection applications.

### 3.4. Leakage Current and Capacitance of a Novel Polysilicon-Fill-Strengthened Etch-Through 3D Trench Electrode Detector

Leakage current is the noise of the detector itself, and this noise level is closely related to the detector material structural design. By optimizing material selection and manufacturing processes (such as reducing interface defects), the leakage current of complex structures can be effectively suppressed due to the reduction in the density of defects. The magnitude of leakage current directly affects the detector performance [21].

The leakage current per unit volume caused by irradiation is directly proportional to the irradiation fluence. For a 1 MeV equivalent neutron irradiation fluence, $\varphi_{n}$, the change in current can be written as:

(2) $$\frac{J}{Vol_{dep}}=\alpha \varphi_{n}$$

where α is the damage coefficient, α = $4\times 10^{-17}A/\mathrm{cm}$, and $Vol_{dep}$ is the volume of the depletion zone [19].

Assuming the effective defect energy level is in the middle of the energy band, the leakage current in the depletion region can be expressed as:

(3) $$J≅J_{gen}=\frac{{en}_{i}Vol_{dep}}{2\tau }$$

where *e* is the basic charge, $n_{i}$ is the intrinsic carrier concentration in silicon ($1.5\times 10^{10}/{\mathrm{cm}}^{3}$), and τ is the minority carrier lifetime [22].

The value of τ obtained from Equations (2) and (3) is:

(4) $$\tau =\frac{{en}_{i}}{2\alpha \varphi_{n}}$$

and will be input into the SRH composite model in our simulation tool for calculating the leakage current of the 3D trench electrode silicon detector after irradiation in our simulations.

**Figure 10:**
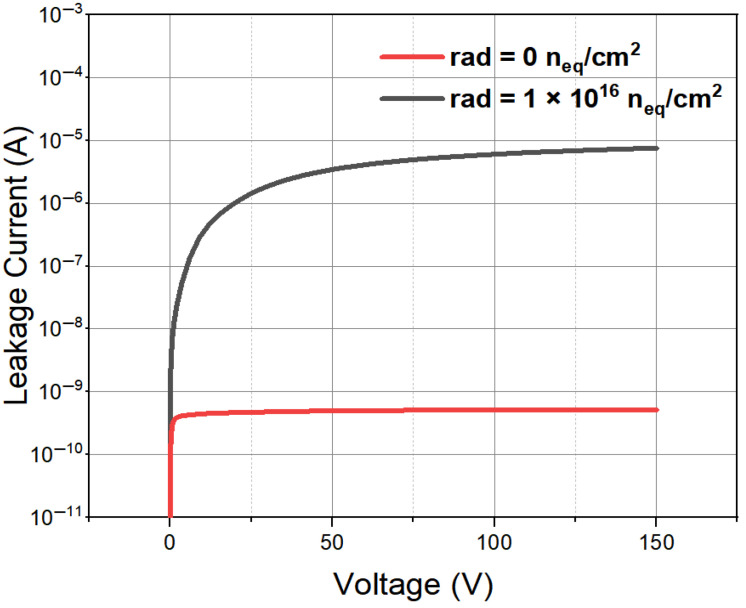
The leakage current curve of a novel polysilicon-fill-strengthened etch-through 3D trench electrode detector under different irradiation environments.

Figure 10 shows the simulated leakage current (with the input of Equation (4) in the simulation model of CONSRH) curves of the novel polysilicon-fill-strengthened etch-through 3D trench electrode detector before irradiation and after a radiation to a fluence of $1\times 10^{16}$ n_eq_/cm^2^, respectively. Here, we use the doping concentration to simulate the space charge induced by radiation effects. The current approximation treatment does not take into account the secondary effects that may be caused by actual deep energy levels, such as temperature dependence and the double junction effect; however, this may not affect the main conclusions of this work. As the detector is irradiated by neutrons and charged particles, the effective doping concentration $N_{eff}$ will increase linearly with 1 MeV neutron-equivalent fluence $\varphi_{neq}$ at high fluences, as (for N-type Si) [23]:

(5) $$N_{eff}≅N_{d0}e^{-\gamma \varphi_{eq}}-\beta \varphi_{eq}$$

where $N_{d0}$ is the initial doping concentration, γ is the donor impurity removal rate, β is the deep level acceptor introduction rate, and $\varphi_{eq}$ is the 1 MeV neutron-equivalent fluence. Since γ is about $1\times 10^{-13}\;{\mathrm{cm}}^{2}$ and β here is 0.01/cm for proton radiation [19], in the case of $\varphi_{eq}$ > $1\times 10^{14}$ n_eq_/cm^2^,

(6) $$N_{eff}≅-\beta \varphi_{eq}$$

From Figure 10, it can be seen that as the voltage increases, the leakage current gradually increases and tends to saturate. For ease of reading, the absolute values of bias voltage and leakage current are displayed. Thus, we obtained a leakage current of $4.8\times 10^{-10}\;A$ for a detector unit (120 µm × 120 µm × 340 µm) with an electrode spacing of 45 µm when fully depleted. As shown in Figure 10, as the radiation fluence increases, both the detector full depletion voltage and the detector saturation leakage current increase accordingly.

In a radiation environment, the lifetime of charge carriers will be significantly shortened. As the carrier lifetime becomes shorter, the leakage current of the detector will increase, thus impacting its overall performance in a significant manner. High radiation conditions not only exacerbate this phenomenon, but also have a negative impact on the detection charge collection efficiency, response speed, and signal integrity of the detector, leading to a degradation in its performance.

**Figure 11:**
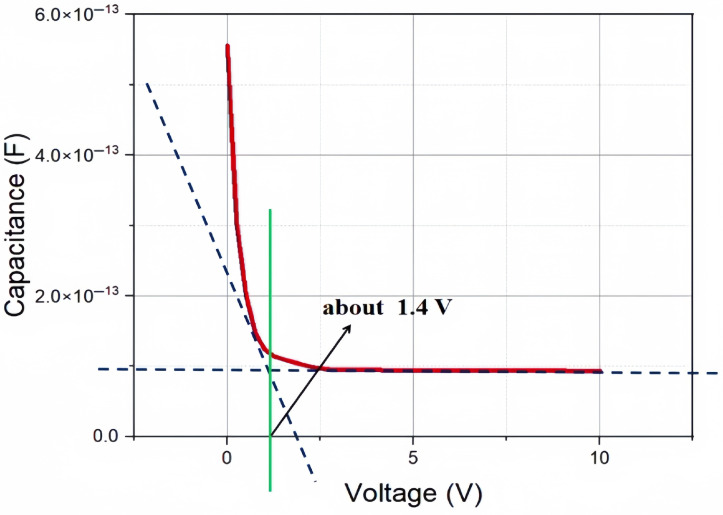
The capacitance curve of a novel polysilicon-fill-strengthened etch-through 3D trench electrode detector without irradiation.

As shown in Figure 11, in an environment without irradiation and low bias voltage, the capacitance of the detector is relatively large. The capacitance is inversely proportional to the depletion depth, and when the depletion depth reaches the length of that of the detector electrode spacing (in 2D cases, it is the detector thickness *d*, and in our 3D case it is *R*) the capacitance value will saturate at the theoretical value of geometric capacitance determined by the geometric structure of the detector (determined by *d* or *R* for 2D or 3D cases, respectively). Figure 11 also confirms the result obtained earlier that the full depletion voltage of the detector is around 1.4 V.

According to the capacitance formula of the 3D trench electrode detector [24],

(7) $$C=\frac{8\epsilon_{r}\epsilon_{0}}{\ln\frac{R}{r_{c}}}\times l$$

where $\epsilon_{r}$ is the relative dielectric constant, *R* is the radius of the detector, $r_{c}$ is the radius of the central cylindrical electrode, and *l* is the length (or etching depth) of the central cylindrical electrode (and therefore it is equal to the detector thickness), the theoretical value of capacitance can be obtained as approximately 106 fF, and the simulation result of 99 fF is close to this theoretical value.

**Figure 12:**
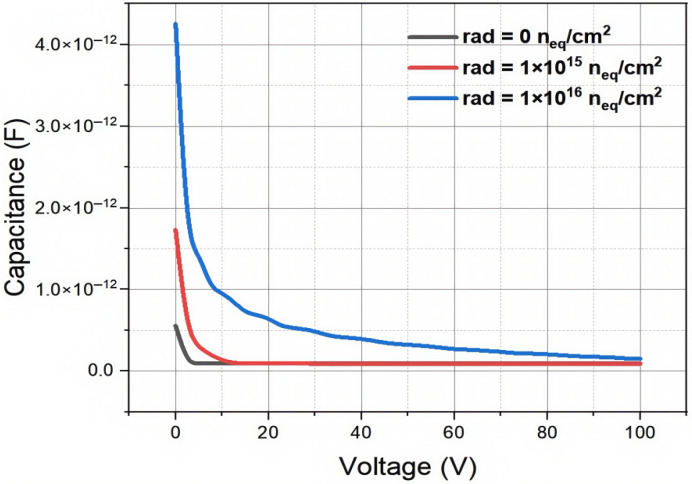
The capacitance curve of a novel polysilicon-fill-strengthened etch-through 3D trench electrode detector under different irradiation environments.

For the capacitance–voltage simulations of irradiated detectors, we used Equation (6) to determine the detector’s effective doping concentration on irradiation. Figure 12 illustrates that when the bias voltage is low, the capacitance is large. As the radiation flux increases, the capacitance of the detector also increases. However, after reaching the fully depleted state, the saturation capacitance of the detector remains unchanged, and the size of the capacitor is mainly determined by the structure of the detector, namely the geometric capacitance. From the simulation results, it can be seen that as the radiation fluence increases, the full depletion voltage of the detector shows an upward trend. This indicates that the increase in radiation fluence has a significant impact on the electrical performance of the detector, leading to an increase in the full depletion voltage, and therefore the detector’s operation voltage. The results demonstrate that even at a extremely high radiation fluence of $1\times 10^{16}$ n_eq_/cm^2^, the full depletion of our new detector is only about 100 Volts, which is entirely operational for the detector. We note that applying 100 V between the two electrodes may need additional insulation layers that may require extra processing steps in the fabrication of the detector.

### 3.5. The Weighting Field of a Novel Polysilicon-Fill-Strengthened Etch-Through 3D Trench Electrode Detector

**Figure 13:**
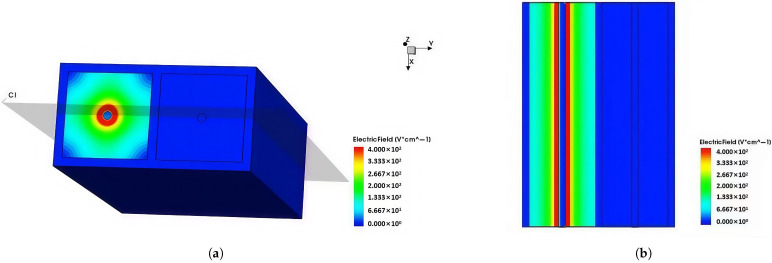
The (**a**) 3D and (**b**) 2D weighting field distributions of a novel polysilicon-fill-strengthened etch-through 3D trench electrode detector.

The detector weighting field is an important parameter for studying the coherence of silicon detector arrays, which is related to the structure of the detector and the selection of the collection electrode. Figure 13a shows the 3D distribution of the weighting field of the detector array, while the 2D distribution obtained through the cross-sectional along the X-axis is shown in Figure 13b. The findings reveal that the weighting field in the neighboring unit is nearly negligible, suggesting minimal crosstalk and very weak coherence between adjacent units. From Figure 13, it can also be seen that the electric field strength at the corners of the square unit is lower than that in the central area, which is due to the asymmetrical square shape deviated from the symmetrical circular shape. In this work, without losing generality, we choose the square shape for simplicity in the simulation process. A more symmetrical hexagonal-shaped detector structure will be studied in the future.

### 3.6. The Transient Induced Current and Charge Collection Efficiency of a Novel Polysilicon-Fill- Strengthened Etch-Through 3D Trench Electrode Detector

**Figure 14:**
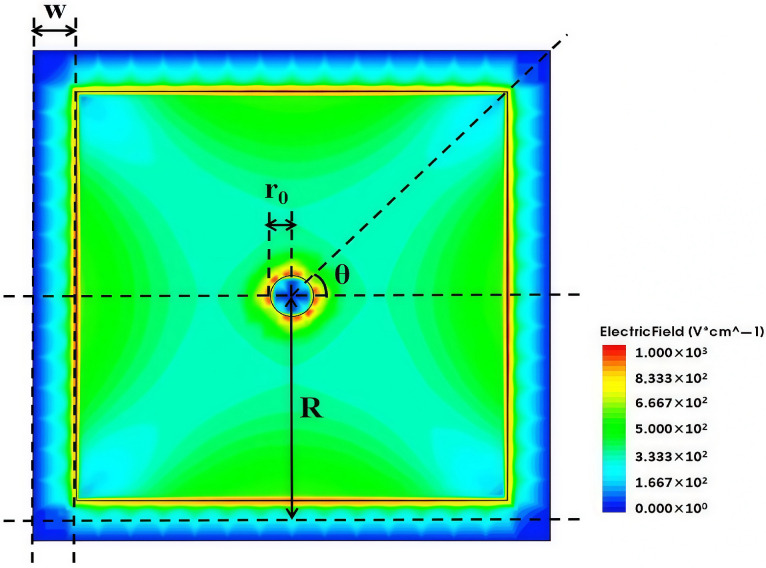
Two-dimensional cross-section of electric field at z = 170 under non-irradiation conditions, V = 1.4 V.

The simulation of transient current signal induced on the detector electrode is based on the fundamental principle of the Shockley–Ramo theorem proposed by William Shockley and Simon Ramo in 1949 [25], which is used to calculate the induced current generated when charged particles incident on to electronic devices, generating electron and hole pairs. From Figure 14, it can be seen that electric field along the diagonal directions are lower than other regions due to the deviation in symmetry of the square shape from that of the circular shape. If the incident point of a particle falls on those diagonal lines with lower electric fields, the charge collection efficiency will be lower than those of other incident points for irradiated detectors. It is worth noting that the best charge collection efficiency is obtained for particle incident points falling along the lines at θ = 0°, 90°, 180°, and 270° [24].

According to the Shockley–Ramo theorem, the induced current for electrons and holes at *t* time is [26,27]:

(8) $$i^{e,h}\left(t\right)=q_{e,h}\cdot \vec{v_{dr}^{e,h}}\cdot \vec{E_{w}}$$

where the charge for electrons or holes *q* = 80 e’s/µm × $d_{eff}$ for the minimum ionization particle incident, $v_{dr}$ is the drift velocity for electrons and holes, $E_{w}$ is the weighting field, obtained from 3D simulation, and $d_{eff}$ is the effective depth of the detector and of the trench electrode; the value of $d_{eff}$ here is 340 µm. The carrier drift velocity $v_{dr}$ (applicable to both electrons and holes) can be mathematically described by incorporating the saturation velocity $v_{s}$ effect through the following expression:

(9) $$v_{\mathrm{dr}}=\frac{dr^{e,h}}{dt}=\frac{u^{e,h}E\left(r^{e,h}\right)}{1+\frac{u^{e,h}E\left(r^{e,h}\right)}{v_{s}}}$$

Here, for both electrons and holes, the approximate value of $v_{s}$ is about $1\times 10^{7}\mathrm{cm}/s$, $u^{e,h}$ is the low field mobility of charge carriers, where electrons are 1450 cm^2^/s/V and holes are 450 cm^2^/s/V, and $E(r^{e,h})$ is the electric field strength at the position $r^{e,h}$ of the drift electron or hole, which is obtained through the three-dimensional electric field simulation of the detector.

**Figure 15:**
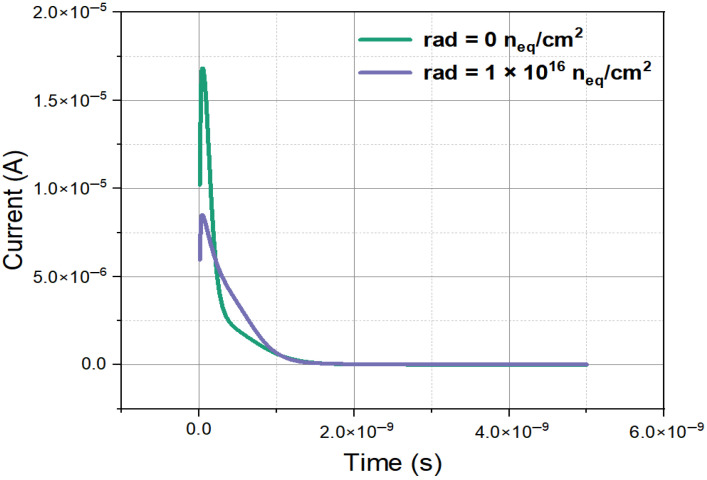
The induced current curve of a novel polysilicon-fill-strengthened etch-through 3D trench electrode detector under different irradiation environments.

In the simulation, we use *q* = 80 e’s/µm (ionization charge density per unit path length) as the physical expression of calculation code of LET_S in the simulation tool. LET_S represents the rate at which particles deposit energy, i.e., linear energy transfer, in silicon in simulation language. In this study, we adopted a value of $1.28\times 10^{-5}$ for LET_S. By using the minimum ionization particle model in the TCAD, we obtained the induced current characteristics in the region of the effective silicon body of the detector with similar methods used in [28], with the results shown in Figure 15. We note that in these transient induced current simulations, Equations (8) and (9) were not actually used, which are only for the purposes of physical explaining. Figure 15 shows the relationship between the detector induced current and drift time before and after MIP incidence at the point of (X = 10, Y = 10, Z = 0) along the Z axis with a bias voltage of 100 V. From the figure, it can be seen that the peak values of the current curve are all within 100 ps, indicating that the detector’s response speed is relatively fast. It can also be seen that the induced current generated inside the detector after irradiation is smaller than the induced current inside the detector under non-irradiation conditions. This is because irradiation can lead to an increase in defects, causing carriers to be trapped during the drift process, and carriers cannot all drift to the electrode, so the induced current of the detector will decrease. By integrating the induced current, we obtained the total charge (about 4 fC).

For irradiated detectors, there will be charge trapping due to radiation-generated defects. The induced current will be reduced in time due to charge trapping [22]:

(10) $$i\left(t\right)=q_{0}\cdot v_{dr}\left(t\right)\cdot E_{w}\cdot e^{-\frac{t}{\tau_{t}}}$$

where $q_{0}$ is the initial charge generated by an incident particle, and $\tau_{t}$ is the trapping constant, ${\tau_{t}}^{-1}$ = $5\times 10^{-7}\varphi_{eq}$. Therefore, the collected charge at time *t* is

(11) $$Q\left(t\right)=\int_{0}^{t}q_{0}\cdot v_{dr}\left(t\right)\cdot E_{w}\cdot e^{-\frac{t}{\tau_{t}}}dt$$

The charge collected for an incident induced charge moving from particle incident point $r_{1}$ to *r* is

(12) $$Q\left(r\right)=q_{0}\cdot \int_{r}^{r_{1}}E_{w}\cdot e^{-\frac{r_{1}-r}{v_{s}\tau_{t}}}dr$$

where for large bias voltages, we have approximated $V_{dr}\left(t\right)$ as the saturation velocity $v_{s}$ = $1\times 10^{7}$ cm/s, and *r* = $r_{1}-v_{s}t$ (*t* = $\frac{r_{1}-r}{v_{s}}$).

To estimate Q(r), we further approximate the weighting field $E_{w}$ as a constant, $E_{w}=\frac{1}{\left(R-r_{0}-\frac{w}{2}\right)}$, without losing physical meaning (the real $E_{w}\left(r\right)$ is $\frac{1}{rln(\frac{R-\frac{w}{2}}{r_{0}})}$, which will significantly complicate the calculations). Equation (12) can be solved and written as:

(13) $$Q\left(r\right)=q_{0}\frac{v_{s}\tau_{t}e^{-\frac{r_{1}}{v_{s}\tau_{t}}}}{R-r_{0}-\frac{w}{2}}\left(e^{\frac{r_{1}}{v_{s}\tau_{t}}}-e^{\frac{r}{v_{s}\tau_{t}}}\right)=q_{0}\frac{v_{s}\tau_{t}}{R-r_{0}-\frac{w}{2}}\left(1-e^{-\frac{r_{1}-r}{v_{s}\tau_{t}}}\right)$$

When $r=r_{0}$, $r_{1}=R-\frac{w}{2}$, the collected charge is:

(14) $$Q=q_{0}\frac{v_{s}\tau_{t}}{R-r_{0}-\frac{w}{2}}\left(1-e^{-\frac{R-r_{0}-\frac{w}{2}}{v_{s}\tau_{t}}}\right)$$

Therefore, the charge collection efficiency is

(15) $$\mathrm{CCE}=\frac{Q}{q_{0}}=\frac{v_{s}\tau_{t}}{R-r_{0}-\frac{w}{2}}\left(1-e^{-\frac{R-r_{0}-\frac{w}{2}}{v_{s}\tau_{t}}}\right)$$

For our case, *R* = 55 µm, $r_{0}$ = 5 µm, *w* = 10 µm, at $\varphi_{eq}$ = $1\times 10^{16}$ n_eq_/cm^2^, $v_{s}\tau_{t}$ = 20 µm, CCE = 39.76%. Therefore, our detector is quite radiation-hard. In reference [24], it was noted by the researchers that charge collection efficiency remains improvable through further minimization of the electrode spacing.

We noticed that there was a recent report on a 3D trench wall electrode (straight) detector showing that even with a extremely high radiation fluence of $1\times 10^{17}$ n_eq_/cm^2^, there was a good timing resolution of 10 ps [29].

### 3.7. Detector Array of a Novel Polysilicon-Fill-Strengthened Etch-Through 3D Trench Electrode Detector

**Figure 16:**
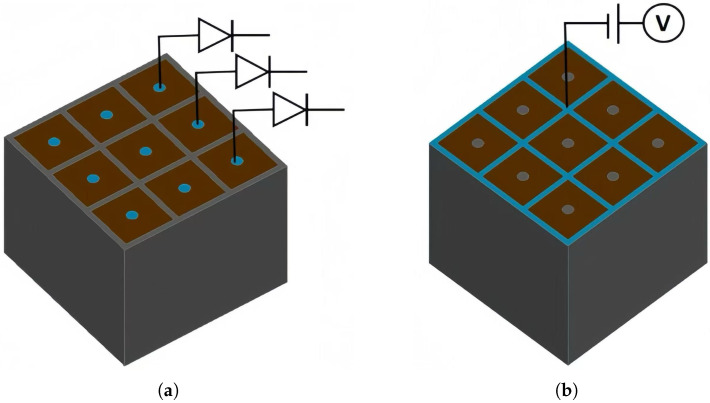
The schematic of (**a**) the top side and (**b**) the bottom side of a novel polysilicon-fill-strengthened etch-through 3D trench electrode detector array.

The novel polysilicon-fill-strengthened etch-through 3D trench electrode detector unit can form a detector array with ultra-fast and ultra-radiation-resistant characteristics. Figure 16a,b is a schematic diagram of a 3 × 3 array, where adjacent detector units are separated by highly doped trenches and share the same trench electrodes, greatly reducing dead zones and significantly improving detector performance. Each detector pixel is connected to a readout channel, which is connected to an application-specific integrated circuit (ASIC).

## 4. Conclusions

After in-depth research on the design and functionality of a new type of 3D trench electrode detector, we conducted detailed and comprehensive simulation studies using Sentaurus TCAD simulation software. We obtained key electrical parameters such as the electron concentration profiles, full depletion voltage, electric potential distribution, electric field distribution, leakage current, capacitance, weighting field distribution, and the induced transient current of the detector. In addition, we also conducted simulation tests and analyses on detectors that had undergone high-fluence irradiation to evaluate their long-term stability and performance degradation.

This new type of detector is etched and filled on both sides of the silicon detector to form through-trench electrodes, which solves the problem of the detector’s main body falling off the detector. Separating the biasing electrode and readout electrode on two opposite sides of the detector may prevent breakdown between the two electrodes caused by discharge between the metal contacts of the two electrodes. In the absence of irradiation, the full depletion voltage of our detector is approximately 1.4 V, which is close to the theoretical calculated value. Our detector has a low capacitance, which is a common characteristic of 3D trench electrode detectors. Calculation results show that our detector can sustain a radiation fluence of up to $1\times 10^{16}$ n_eq_/cm^2^, with a CCE of about 40%.

For pixillated detectors, the simulation results show that our units in the detector are isolated from each other due to etch-through highly doped trenches, eliminating the electronic interference between them.

## Data Availability

The original contributions presented in the study are included in the article, further inquiries can be directed to the corresponding author.
